# The Value of Blood Urea Nitrogen in the Prediction of Risks of Cardiovascular Disease in an Older Population

**DOI:** 10.3389/fcvm.2021.614117

**Published:** 2021-05-20

**Authors:** Qin Lan, Liang Zheng, Xiaohui Zhou, Hong Wu, Nicholas Buys, Zhongmin Liu, Jing Sun, Huimin Fan

**Affiliations:** ^1^Shanghai East Hospital, Tongji University, Shanghai, China; ^2^School of Medicine, Tongji University, Shanghai, China; ^3^GaoHang Community Hospital, Pudong New Area, Shanghai, China; ^4^Menzies Health Institute Queensland, Griffith University, Gold Coast, QLD, Australia; ^5^School of Medicine, Griffith University, Gold Coast, QLD, Australia

**Keywords:** blood urea nitrogen, cardiovascular disease, prediction, older populations, risk factors

## Abstract

**Background:** High blood urea nitrogen (BUN) is associated with adverse outcomes in patients with cardiac disease risks. However, no study has explored whether BUN can predict the risk of cardiovascular disease (CVD) in the healthy older population. This study aims to explore the incidence and risk factors of CVD among a healthy older population community in China.

**Design and Methods:** This study was designed as a cohort study with a 4-year follow-up. We recruited 5,000 older people among 137,625 residents of the Gaohang community. In the baseline, subjects were asked to participate in medical screening and biological tests, and answered survey questions. During the follow-up period (2014–2017), the researchers regularly tested the subjects' indicators and assessment scales. We monitored the occurrence of CVD and explored the relationship between BUN and CVD via a Cox regression analysis.

**Results:** During the follow-up, subjects were newly diagnosed with CVD including heart failure (HF), heart disease events, atrial fibrillation, diabetes, hypertension, metabolic syndrome, and kidney disease. The Cox regression analysis found an association between baseline BUN and incident CVD in female subjects, with higher BUN associated with increased risk of AF in females and kidney disease in both male and females. No association was found between BUN and CVD in male subjects.

**Conclusions:** Current results indicate that BUN is a valuable predictive biomarker of CVD. A higher BUN level (>13.51 mg/dL) is associated with an increased occurrence of HF but a decreased occurrence of diabetes and metabolic symptoms in normal older females.

## Introduction

Cardiovascular disease (CVD) is a global public health problem associated with adverse outcomes (1). With economic development and the change of lifestyle, the incidence of CVD in China is rising rapidly. The global prevalence of CVD nearly doubled from 271 million in 1990 to 523 million in 2019, and the number of CVD deaths steadily increased from 12.1 million in 1990, reaching 18.6 million in 2019 (2). It is estimated that China has one of the highest CVD death rates in the world, with one in five adults in China experiencing a cardiovascular disease (3). It is therefore crucial to find biomarkers that can predict the occurrence of CVD so prevention measures can be implemented.

Studies have shown that renal function markers, including blood urea nitrogen (BUN), glomerular filtration rate and creatinine, are associated with mortality from CVD (4, 5). BUN measures the amount of nitrogen in blood that comes from the waste product urea. BUN levels often rise when kidneys are not able to remove urea from the blood normally and a BUN test is used to measure kidney function. Clinical studies have consistently shown that BUN levels are high in patients with CVD and this biomarker is therefore an important independent predictor of outcomes regarding CVD compared with other markers of renal function (6–9). Researchers point out that BUN levels are not only affected by kidney function, but also by endocrine disorders. BUN is a marker of neurohumoral activity and renal function, and thus, can reflect the pathophysiological process of CVD. In addition, BUN can also reflect the relationship between nutritional status, protein metabolism and renal function, and is an important marker of metabolic diseases, nutritional status of patients, and other diseases (10–12).

Studies have demonstrated that a higher BUN level is associated with adverse outcomes in patients with cardiac problems. However, no study has explored whether BUN can predict the risk of occurrence of CVD in a healthy older population in China. We hypothesise that higher BUN levels are a risk factor for CVD in the healthy older population. To test this hypothesis, we designed a prospective cohort study in Gaohang community district located in Pudong New Territory in Shanghai Municipal City whereby data was collected to determine the association between BUN and the incident CVD in a healthy older population.

## Design and Methods

This study was designed as a cohort study with a 4-year follow-up. We recruited 5,000 older Gaohang community members from among the 137,625 residents who participated in health screening at Shanghai East Hospital from 1 March to 30 June 2013. Subjects were included according to the following criteria: (1) Age ≥60 years, (2) Resident of Gaohang community, and (3) conscious and able to write. The exclusion criteria included (1) No current established CVD or other severe disease, (2) Without available BUN data, (3) Severe psychiatric disease, and (4) At a high risk of suicide. At baseline, subjects were asked to participate in medical screening and biological tests, and answered survey questions. During the follow-up period from 2014 to 2017, the researchers regularly tested subjects' indicators and assessment scales. The study was approved by the Ethics Committee of Shanghai East Hospital and all subjects provided consent for their participation in the study.

We used a questionnaire to collect data on the demographic characteristics of the subjects, as well as lifestyle characteristics such as smoking and drinking. Subjects were weighed via a scale during a health examination, and body mass index (BMI) was calculated according to the usual formula [weight (kg) divided by the square of height (m) (kg/m^2^)]. Data on subjects' exercise were collected by questionnaire, and were divided into three levels: no exercise, mild exercise, and moderate exercise. Smoking and alcohol consumption were also investigated, with the threshold for smoking set at least one cigarette per day for at least 1 year, and that for alcohol set at average daily alcohol consumption of more than 50 grammes for at least 1 year.

Blood parameters including glucose, total cholesterol (TC), triglycerides (TG), high-density lipoprotein (HDL), low-density lipoprotein (LDL), BUN, serum creatinine, and C-reactive protein (CRP) were collected, with subjects asked to fast for at least 12 h before collection. Blood pressure was measured with a calibrated sphygmomanometer at resting state. Blood pressure was measured three times, with the interval between measurements 1–2 min. The average of these three measurements was considered the final value of blood pressure.

We monitored the occurrence of diseases during the data collection period, including HF, hypertension, atrial fibrillation, metabolic syndrome, diabetes, and kidney disease. According to the 2013 guidelines of the European Society of Hypertension (ESH) and the European Society of Cardiology (ESC) and the 2010 Chinese guidelines for the management of hypertension, the criteria for hypertension are systolic blood pressure (SBP) ≥140 mmHg and/or diastolic blood pressure (DBP) ≥ 90 mmHg (13, 14). Diagnoses of heart failure, heart disease events, atrial fibrillation, diabetes, hypertension, metabolic syndrome, and kidney disease were confirmed by cardiac specialists from the pathological diagnosis, ECG and ultrasound tests and based on American Heart Association guidelines (1). Metabolic syndrome was defined based on IDF standard with additional Chinese criteria that men had waist circumference 90 cm and more and women with 80 cm and more waist circumference (15). Kidney disease is defined based on eGFI level. The following formulas were used to calculate eGFR by gender: for males, eGFR (mL/min/1.73 m^2^) = 194 × [age]^−0.287^ × [serum creatinine (mg/dL)]^−1.094^; and for females, eGFR (mL/min/1.73 m^2^) = 194 × [age]^−0.287^ × [serum creatinine (mg/dL)]^−1.094^ × 0.739 (16, 17). A value below 60 mL/min/1.73 m2 suggests loss of kidney function and this was confirmed by blood test and medical doctor in the present study. Diabetes was defined based on China criteria of fasting glucose level at 7.0 mmol/L or more (18). Of the 5,000 subjects recruited 4,219 completed the study when the study in 2017.

### Statistical Analysis

Data analysis was performed using SPSS 25.0. Numeric data are expressed as mean ± SD, while categorical data are expressed as numbers and percentages. The chi-squared (χ^2^) test was used to evaluate the differences in the frequency of characteristics including age, occupation, education, marital status, income, BMI, smoking, drinking, and exercise between the diseased and non-diseased group in the 2017 follow-up data. A *t*-test was used to test the difference between the diseased and non-diseased groups for the variables of waist circumference, SBP, DBP, TC, LDL, HDL, glucose, UC, UN, and CRP in the 2017 follow-up data. Chi-square (χ^2^) and *t*-tests were conducted for both male and female participant groups. BUN at baseline was further divided into three quartiles, and the association between BUN and each laboratory-based measure was analysed by using one-way analysis of variances (ANOVA) to examine whether the laboratory-based measures increased with increasing BUN levels in the male and female groups. The prediction of BUN on each disease was conducted by using a Cox regression analysis for the male and female subject groups, with *p* < 0.05 considered statistically significant.

## Results

### Baseline Characteristics and CVD Incidence During the Follow-Up

Baseline indices included age, occupation, education, marital status, monthly income, BMI smoking, drinking, exercise, waist circumference, blood pressure, TC, HDL, LDL, glucose, BUN, uric acid, and CRP. The mean age of all subjects was 72.12 (SD 6.675) for females and 71.75 (SD 6.449) for males. Mean BUN was 5.723 mmol/l (SD: 1.665) in men and 5.567 mmol/l (SD: 1.566) in women in 2013, and 5.723 mmol/l (SD: 1.644) in mean and 5.561 mmol/l (SD: 1.514) in women in 2017. After 4 years' follow-up until 2017, newly diagnosed CVD diseases including HF, heart disease events, atrial fibrillation, diabetes, hypertension, metabolic syndrome, and kidney disease were presented (see Table 1). We compared the differences in baseline characteristics between older people with and without CVD in 2013.

**Table 1:** Comparison of baseline characteristics among subjects by disease (2013).

**Variables**	**Male**			**Female**		
	**Heart disease**	**No heart disease**	**χ^2^t/F(p)**	**Heart disease**	**No heart disease**	**χ^2^t/F(p)**
**Age:** ***n*** **(%)**						
≤ 65 years	63 (17.9)	204 (20.4)	7.93 (0.09)	59 (12.9)	271 (20.3)	19.69 (**0.001**)
66–70 years	113 (32.1)	365 (36.4)		155 (33.8)	455 (34.1)	
71–75 years	74 (21.0)	190 (19.0)		89 (19.3)	242 (18.2)	
76–80 years	70 (19.9)	144 (14.3)		85 (18.5)	231 (17.3)	
≥81 years	32 (9.1)	99 (9.9)		71 (15.5)	134 (10.1)	
**Occupation:** ***n*** **(%)**						
Office worker	75 (21.4)	229 (22.9)	0.53 (0.91)	46 (10.1)	131 (9.8)	1.14 (0.77)
Operator	184 (52.4)	506 (50.6)		115 (25.1)	307 (23.1)	
Farmer	66 (18.8)	186 (18.6)		273 (59.6)	829 (62.3)	
Other	26 (7.4)	79 (7.9)		24 (5.2)	64 (4.8)	
**Education:** ***n*** **(%)**						
Primary below	22 (6.3)	52 (5.2)	3.18 (0.37)	157 (34.2)	381 (28.7)	8.67 (**0.03**)
Primary	74 (21.0)	181 (18.1)		117 (25.5)	310 (23.3)	
Secondary school	206 (58.5)	596 (59.5)		165 (35.9)	575 (43.3)	
College or above	50 (14.2)	172 (17.2)		20 (4.4)	63 (4.7)	
**Marital:** ***n*** **(%)**						
No married	38 (10.8)	109 (10.9)	0.00 (0.95)	151 (33.0)	410 (30.9)	0.69 (0.41)
Married	313 (89.2)	887 (89.1)		307 (67.0)	918 (69.1)	
**Monthly income:** ***n*** **(%)**						
<1,500 yuan	58 (16.6)	142 (14.4)	1.42 (0.70)	160 (35.1)	477 (36.4)	1.13 (0.77)
1,500–2,000 yuan	63 (18.1)	191 (19.4)		85 (18.6)	221 (16.9)	
2,000–2,500 yuan	88 (25.2)	238 (24.2)		107 (23.5)	296 (22.6)	
>2,500 yuan	140 (40.1)	414 (42.0)		104 (22.8)	316 (24.1)	
Body mass index: M (SD)	24.63 (3.29)	24.41 (2.99)	1.09 (0.28)	25.03 (3.79)	24.60 (3.48)	2.12 (**0.03**)
**Body mass index:** ***n*** **(%)**						
BMI <18.5	8 (2.3)	27 (2.7)	5.31 (0.26)	16 (3.5)	39 (2.9)	9.53 (**0.05**)
18.5–23.9	138 (39.2)	421 (42.0)		165 (35.9)	567 (42.5)	
24.0–27.9	159 (45.1)	446 (44.5)		188 (41.0)	532 (39.9)	
28.0–32.0	40 (11.4)	101 (10.1)		68 (14.8)	151 (11.4)	
BMI>32	7 (2.0)	7 (0.7)		22 (4.8)	44 (3.3)	
**Smoking:** ***n*** **(%)**						
No	176 (50.0)	489 (48.8)	0.31 (0.86)	446 (97.1)	1,314 (98.6)	
Smoking	108 (30.7)	306 (30.5)		9 (2.0)	12 (0.9)	
Smoked before, but stopped	68 (19.3)	207 (20.7)		4 (0.9)	7 (0.5)	
**Drinking:** ***n*** **(%)**						
No	226 (64.2)	611 (61.0)	2.30 (0.32)	449 (97.8)	1,310 (98.2)	
Drank, but stopped	33 (9.4)	84 (8.4)		4 (0.9)	1 (0.1)	
Drinking	93 (26.4)	307 (30.6)		6 (1.3)	22 (1.7)	
**Exercise:** ***n*** **(%)**						
No exercise	19 (5.5)	29 (3.0)	5.74 (0.06)	32 (7.2)	64 (5.1)	2.99 (0.22)
Light-intensity	213 (62.1)	646 (66.9)		313 (70.2)	890 (70.6)	
Moderate-intensity	111 (32.4)	291 (30.1)		101 (22.6)	307 (24.3)	
Waist circumference: M (SD)	88.00 (9.67)	87.67 (8.69)	0.56 (0.57)	86.18 (9.03)	85.28 (9.07)	1.85 (0.07)
Diastolic blood pressure: M (SD)	83.11 (8.85)	82.06 (9.01)	1.89 (0.06)	81.51 (8.44)	81.32 (8.52)	0.42 (0.68)
Systolic blood pressure: M (SD)	139.17 (17.01)	137.66 (17.18)	1.42 (0.16)	139.76 (17.05)	139.37 (17.24)	0.41 (0.68)
TC (mmol/L): M (SD)	4.70 (0.89)	4.74 (0.90)	−0.71 (0.48)	5.16 (0.90)	5.18 (0.94)	−0.48 (0.63)
HDL (mmol/L): M (SD)	1.37 (0.39)	1.38 (0.38)	−0.50 (0.62)	1.56 (0.42)	1.52 (0.39)	1.68 (0.09)
LDL (mmol/L): M (SD)	3.11 (0.80)	3.14 (0.84)	−0.53 (0.60)	3.41 (0.83)	3.46 (0.87)	−1.15 (0.25)
Glucose (mmol/L): M (SD)	5.59 (1.71)	5.64 (1.77)	−0.54 (0.59)	5.82 (1.81)	5.76 (1.81)	0.68 (0.50)
Urea Nitrogen (mmol/L): M (SD)	5.62 (1.58)	5.58 (1.41)	0.43 (0.66)	5.69 (1.57)	5.50 (1.51)	2.35 (**0.02**)
Uric Acid (mg/dL): M (SD)	5.96 (1.35)	5.97 (1.41)	−0.12 (0.90)	5.25 (1.38)	1.98 (3.17)	2.27 (**0.02**)
CRP (mg/L): M (SD)	2.40 (5.07)	1.97 (4.12)	1.43 (0.15)	1.98 (3.17)	2.26 (5.24)	−1.37 (0.17)

*TC, total cholesterol; HDL, high-density lipoprotein; LDL, low-density lipoprotein; CRP, C-reactive protein; TG, cr. Bold values refers to statistically significant*.

Heart failure occurred in 352 males and 459 females, with the highest incidence of seven diseases because we included borderline heart failure patients with ejection fraction (EF) score between 40 and 60 using ultra sound. Comparing male subjects with and without HF, there was no difference (*p* > 0.05) in age, occupation, education, marital status, monthly income, BMI, smoking, drinking, exercise, waist circumference, blood pressure, TC, HDL, LDL, glucose, BUN, uric acid, or CRP. However, there were significant differences in age (*F* = 19.69, *p* = 0.001), education (*F* = 8.67, *p* = 0.03), BMI (*t* = 2.12, *p* = 0.03), BUN (*t* = 2.35, *p* = 0.02), and uric acid (*F* = 2.35, *p* = 0.02).

Heart disease events occurred in 142 males and 229 females. There was no difference (*p* > 0.05) in age, occupation, education, marital status, monthly income, BMI, smoking, drinking, exercise, waist circumference, blood pressure, TC, HDL, LDL, glucose, BUN, uric acid, or CRP between female subjects who experienced or did not experience a heart disease event. However, there were significant differences in education (*F* = 8.96, *p* = 0.03), smoking (*F* = 9.37, *p* = 0.009), waist circumference (*F*=-2.40, *p* = 0.02), and systolic blood pressure (*F* = 2.70, *p* = 0.007).

Atrial fibrillation occurred in 55 males and 77 females. There were no differences (*p* > 0.05) in baseline characteristics between male subjects with and without atrial fibrillation. However, there were differences in age, monthly income and exercise between female subjects with and without atrial fibrillation (*p* < 0.05).

Diabetes occurred in 98 males and 135 females. BMI was higher in subjects with diabetes than those without diabetes in the male group (*t* = 2.73, *p* = 0.007), and there were significant differences in BMI, glucose, and uric acid between female subjects with and without diabetes.

Hypertension occurred in 249 males and 360 females. There are significant differences (*p* < 0.05) in BMI, DBP, SBP, and HDL between male subjects with and without hypertension. Female subjects with hypertension also showed lower baseline SBP and DBP than those without hypertension (*t* = −12.06, *p* < 0.001; *t* = −14.08, *p* < 0.001).

Metabolic syndrome occurred in 175 males and 214 females. In the male group, there were significant differences (*p* < 0.05) in occupation, BMI and SBP between subjects with and without metabolic syndrome. In the female group, there were significant differences in age, occupation, monthly income, BMI, waist circumference, DBP, SBP, glucose, BUN, and uric acid between subjects with and without metabolic syndrome (*p* < 0.05).

Kidney disease occurred in 163 males and 221 females. The male subjects with kidney disease showed lower baseline HDL than those without kidney disease (*t* = −2.28, *p* = 0.02). There were differences in age, occupation, and BUN between female subjects with and without kidney disease (*p* < 0.05).

### Comparison Among Characteristics by Category of Baseline BUN

As shown in Table 2, according to the three levels of baseline BUN (≤0.10–5.90 mmol/L, 5.91 mmol−7.00 mmol/L, 8.0 or more mmol/L), male and female subjects were divided into three groups, respectively. The male and female groups both showed significant differences in age among the three groups (*F* = 18.49, *p* < 0.001; *F* = 28.79, *p* < 0.001). Of males, the group with higher BUN showed higher TC, HDL, glucose, HbA1c, and creatinine than the group with low BUN (*p* < 0.05). Of females, there were also significant differences in TC, triglyceride, LDL, glucose, HbA1c, and creatinine among the three groups. Increasing levels BUN were associated with increasing level of TC, triglyceride, LDL, Glucose, HbA1c, and creatinine.

**Table 2:** Comparison between characteristics by category of baseline.

**Variables**	**Urea nitrogen tertile (mmol/L) (Male)**				**Urea nitrogen tertile (mmol/L) (Female)**			
	**0.10–5.90**	**5.90–7.99**	**8.0 and more**	**F(p)**	**0.10–5.90**	**5.90–7.99**	**8.0 and more**	**F(p)**
**Age: M(SD)**	70.79 (5.97)	71.34 (6.46)	72.96 (6.64)	18.49 (** <0.001**)	71.05 (6.19)	71.84 (6.48)	73.65 (7.14)	28.79 (** <0.001**)
Body mass index: M(SD)	24.51 (3.00)	24.60 (3.14)	24.35 (3.20)	0.97 (0.38)	24.67 (3.47)	24.76 (3.65)	24.74 (3.68)	0.15 (0.86)
Waist circumferences (cm):M(SD)	88.01 (8.59)	88.16 (8.82)	87.77 (9.27)	0.29 (0.75)	85.09 (9.45)	85.67 (8.86)	86.24 (9.35)	2.77 (0.06)
Systolic blood pressure (mmHg):M(SD)	137.94 (17.41)	138.47 (17.00)	138.40 (17.62)	0.15 (0.86)	139.15 (17.00)	138.57 (17.26)	140.16 (17.61)	1.56 (0.21)
Diastolic blood pressure (mmHg):M(SD)	82.92 (8.87)	82.53 (8.67)	82.12 (9.74)	1.11 (0.33)	81.31 (8.52)	81.87 (8.76)	80.80 (8.73)	2.77 (0.06)
Total cholesterol (mmol/L):M(SD)	4.61 (0.89)	4.78 (0.89)	4.80 (0.95)	7.74 (** <0.001**)	5.10 (0.98)	5.23 (0.98)	5.25 (0.95)	4.98 (**0.007**)
Triglyceride (mmol/L):M(SD)	1.56 (0.98)	1.55 (1.22)	1.50 (1.06)	0.61 (0.54)	1.81 (1.55)	1.66 (0.96)	1.65 (1.01)	4.02 (**0.02**)
HDL (mmol/L):M(SD)	1.34 (0.37)	1.38 (0.39)	1.43 (0.39)	6.57 (**0.001**)	1.48 (0.37)	1.54 (0.40)	1.56 (0.41)	9.19 (** <0.07**)
LDL (mmol/L):M(SD)	3.06 (0.80)	3.18 (0.83)	3.15 (0.88)	3.10 (**0.05**)	3.38 (0.85)	3.49 (0.90)	3.48 (0.89)	3.43 (**0.03**)
Glucose (mmol/L):M(SD)	5.40 (1.58)	5.70 (1.77)	5.90 (2.09)	10.96 (** <0.001**)	5.63 (1.60)	5.82 (1.94)	6.06 (2.10)	9.28 (** <0.001**)
HbA1c (%):M(SD)	6.14 (0.89)	6.35 (1.14)	6.41 (1.21)	9.48 (< **0.001**)	6.32 (1.00)	6.40 (1.17)	6.48 (1.13)	3.97 (**0.02**)
Creatinine (umol/L):M(SD)	79.90 (12.89)	83.70 (14.48)	94.09 (26.31)	86.46 (< **0.001**)	63.28 (11.48)	66.90 (13.70)	76.42 (25.04)	107.20 (** <0.001**)
CRP (mg/L):M(SD)	2.25 (4.45)	2.21 (4.85)	2.05 (3.89)	0.34 (0.72)	2.62 (6.74)	2.11 (3.87)	2.19 (4.10)	2.21 (0.11)

*HDL: high-density lipoprotein; LDL: low-density lipoprotein; CRP: C-reactive protein. Bold values refers to statistically significant*.

### Cox Regression Analysis for Cardiovascular Diseases

With CVD related disease as the dependent variable and the BUN categories as the independent variable, Cox regression analysis was used to evaluate the association between the level of BUN and CVD. The Cox regression analysis showed that abnormal level of baseline BUN was associated with an increased risk of disease in both men with HR 1.74 (95% CI: 0.99–3.06, *P* < 0.05) and women with HR 1.86 (95% CI: 1.19–2.92, *P* < 0.01). No association was found between BUN and occurrence of heart failure, metabolic syndrome, diabetes, and heart disease events (see Table 3).

**Table 3:** Risk of disease by categories of serum urea nitrogen of baseline (raw percentages).

**Variables**	**Urea nitrogen tertile (mmol/L) (Male)**			**Urea nitrogen tertile (mmol/L) (Female)**		
	**0.10–5.90**	**5.90–7.99**	**8.0 and more**	**0.10–5.90**	**5.90–7.99**	**8.0 and more**
**Heart failure**						
No. of subjects: *n* (heart failure subjects)	246 (53)	243 (60)	221 (67)	340 (87)	335 (103)	250 (77)
No. of incidents of HF (rate: %)	113 (10.76)	123 (11.65)	116 (11.37)	135 (8.82)	174 (11.61)	105 (8.87)
Person-years	1,050	1,056	1,020	1,531	1,499	1,184
Adjusted 95% CI						
**Heart disease events**						
No. of subjects: *n* (HD events subjects)	268 (163)	267 (153)	239 (152)	367 (228)	356 (221)	261 (171)
No. of incidents of HD event (rate: %)	42 (3.44)	53 (4.37)	47 (4.15)	78 (4.68)	76 (4.70)	75 (5.91)
Person-years	1,222	1,214	1,132	1,668	1,616	1,268
Adjusted 95% CI						
**Atrial fibrillation**						
No. of subjects: *n* (atrial fibrillation subjects)	449 (18)	193 (7)	32 (0)	607 (20)	210 (8)	44 (5)
No. of incidents of AF (rate: %)	18 (1.336)	7 (1.209)	0 (0)	20 (1.098)	8 (1.270)	5 (3.789)
Person-years	1,347	579	96	1,821	630	132
Adjusted 95% CI	1	0.91 (0.38–2.17)	0	1	1.16 (0.51–2.63)	3.45 (1.29–9.19)^**^
**Diabetes**						
No. of subjects: *n* (diabetes subjects)	263 (58)	258 (70)	231 (73)	361 (104)	346 (102)	261 (78)
No. of incidents of DM (rate: %)	36 (2.97)	34 (2.77)	28 (2.48)	55 (3.27)	46 (2.79)	34 (2.60)
Person-years	1,212	1,226	1,131	1,683	1,646	1,308
Adjusted 95% CI						
**Hypertension**						
No. of subjects: *n* (hypertension subjects)	258 (112)	258 (115)	225 (115)	352 (163)	344 (162)	248 (124)
No. of incidents of hypertension (rate: %)	79 (6.85)	90 (7.85)	80 (7.53)	125 (7.86)	123 (7.89)	112 (9.51)
Person-years	1,153	1,147	1,063	1,591	1,558	1,178
Adjusted 95% CI						
**Metabolic syndrome**						
No. of subjects: *n* (Mt Syndrome subjects)	246 (98)	248 (109)	207 (86)	332 (240)	324 (231)	236 (161)
No. of incidents of Mt Syndrome (rate: %)	57 (4.99)	64 (5.53)	54 (5.20)	95 (6.03)	62 (3.99)	57 (4.71)
Person-years	1,142	1,158	1,039	1,575	1,554	1,210
Adjusted 95% CI						
**Kidney disease**						
No. of subjects: *n* (kidney disease subjects)	868 (93)	411 (56)	77 (14)	1,212 (141)	482 (58)	106 (22)
No. of incidents of kidney disease (rate: %)	93 (4.19)	56 (5.54)	14 (7.37)	141 (4.62)	58 (5.07)	22 (8.90)
Person-years	2,218	1,010	190	3,054	1,145	247
Adjusted 95% CI	1	1.30 (0.94–1.81)	1.74 (0.99–3.06)^*^	1	1.07 (0.79–1.45)	1.86 (1.19–2.92)^**^

*Metabolic syndrome confounding factor: Age; CI, confidence interval; eGFR, estimated glomerular filtration rate*.

* *P < 0.05;*

** *P < 0.01*.

*Only significant results in cox regression analysis were presented in the table. Bold values refers to statistically significant*.

## Discussion

This cohort study observed the occurrence of CVD and its association with BUN in a normal older community in China. During a 4-year follow-up, subjects had newly diagnosed CVD including HF, a heart disease event, atrial fibrillation, diabetes, hypertension, metabolic syndrome and kidney disease. We found higher baseline BUN (>13.51 mg/dL) is associated with the increased occurrence of AF in females, and kidney disease in both male and female subjects.

CVD, as a common chronic disease, has become an important public health issue in China. AF is the result of further development of various CVD risk factors and its incidence is increasing in recent years, especially among the older population (19, 20). This study monitored the incidence of CVD, including HF, a heart disease event, atrial fibrillation, diabetes, hypertension, metabolic syndrome, and kidney disease among older people in one community, and found the incidence of HF was the highest among these seven diseases. The results show that effective monitoring and prevention of CVD in the older population are of great significance for public health.

BUN is a metabolite produced by protein digestion and decomposition, which is converted to urea in the liver through urea circulation and then filtered out by glomeruli. Its concentration represents the balance between urea production and renal excretion (7). The complex interaction between heart and kidneys plays an important role in the occurrence and development of CVD. Amir Kazory systematically reviewed 10 articles and demonstrated that higher BUN is associated with worse outcomes for patients with CVD, including acute decompensation and chronic stable CVD. Researchers argue that BUN may be a marker of neurohormone-activated “renal response,” thus participating in the pathophysiological process of CVD (9). Based on previous research findings, we speculate that elevated BUN levels increase plasma osmotic pressure and lead to decreased blood flow, which may be one of the causes of kidney disease (21–24). No study has explored whether BUN is associated with the occurrence of CVD in a healthy population. Our study found female subjects with higher baseline BUN (>13.51 mg/dL) had a higher risk of AF than those with lower BUN. This indicates that BUN is also a risk factor for AF in healthy older people.

The main limitations of this study were its cohort design and observational nature. During the 4-year follow-up, some subjects dropped out of the study. Subjects with higher BUN showed higher levels of TC, HDL, LDL, glucose, HbA1c, and creatinine, which are also risk factors for CVD, so it is not clear whether these factors together induced the CVD, although they were treated as confounding factors in the analysis. In addition, this study did not assess sufficient variables that can alter BUN levels, such as a high protein diet, medication usage and muscle wasting. Thus, a better designed randomised controlled trial is needed to further prove the current findings in the future.

Current results indicate that BUN is a valuable prediction biomarker of CVD. Higher BUN (>13.51 mg/dL) is associated with the increased occurrence of AF in females and Kidney disease in both males and females. As BUN is a commonly used clinical indicator, it is easy to obtain; thus, it is feasible to use BUN as a predictor of AF and kidney disease in community populations.

## Data Availability Statement

The raw data supporting the conclusions of this article will be made available by the authors, without undue reservation.

## Ethics Statement

The studies involving human participants were reviewed and approved by Shanghai East Hospital. The patients/participants provided their written informed consent to participate in this study.

## Author Contributions

HF and ZL conceptualised and designed the study. QL, LZ, XZ, and HW collected data. JS analysed data, drafted, and edited manuscript. NB reviewed and commented on the manuscript. All authors reviewed the manuscript and agreed the paper to be submitted.

## Conflict of Interest

The authors declare that the research was conducted in the absence of any commercial or financial relationships that could be construed as a potential conflict of interest.
